# Predicting extubation readiness by monitoring the electrical activity of the diaphragm after prolonged mechanical ventilation: a pediatric case report

**DOI:** 10.1186/s40981-018-0213-y

**Published:** 2018-10-22

**Authors:** Yusuke Naito, Yoshiyuki Shimizu, Takeshi Hatachi, Yu Inata, Kazue Moon, Kazuya Tachibana, Muneyuki Takeuchi

**Affiliations:** 1 Department of Anesthesiology, Osaka Women’s and Children’s Hospital, 840, Murodo-cho, Izumi, Osaka Japan; 2 Department of Critical Care, Osaka Women’s and Children’s Hospital, 840, Murodo-cho, Izumi, Osaka Japan

**Keywords:** Electrical activity of the diaphragm (Edi), Neuroventilatory efficiency (NVE), Diaphragm function

## Abstract

**Background:**

The intensity of the electrical activity of the diaphragm (Edi) correlates with inspiratory effort. The ratio of tidal volume to the Edi is known as neuroventilatory efficiency (NVE) and is used as an index for ventilation efficiency. Here, we present a case showing that Edi and NVE may be effective parameters to predict successful extubation.

**Case presentation:**

A 6-month-old female infant required prolonged mechanical ventilation after cardiac surgery. Fifty-two days after surgery, her trachea was extubated but required reintubation. Edi monitoring was initiated to assess diaphragm function. The Edi was > 70 mcV just after the reintubation, and her NVE was 1.0 mL/mcV, but gradually decreased. On day 59, her Edi values during the spontaneous breathing trials were 13 mcV with the improvement of NVE (2.5 mL/mcV) and her trachea was extubated without complications.

**Conclusions:**

The Edi and NVE were valuable for deciding the extubation readiness in a long-term mechanically ventilated patient.

## Background

Prolonged mechanical ventilation is associated with nosocomial pneumonia, iatrogenic lung injury, excessive sedation, and unplanned extubation accounting for increased mortality [1]. On the other hand, premature extubation is associated with increased reintubation rate and prolonged length of stay in the intensive care unit (ICU), which in turn accounts for increased mortality [2]. Therefore, it is important to extubate the patient at an appropriate time point that is neither too early nor too late. To achieve this goal, two important factors regarding respiratory function should be considered. One is the improvement of respiratory mechanics. The other is the diaphragm function after extubation. While respiratory mechanics can be objectively evaluated by compliance of the respiratory system (Crs) [1], a standard method for the clinical assessment of diaphragm function has not been established. Diaphragm sonography has been proposed recently, but the conflicting results so far reported carry a substantial risk of misinterpretation [3]. Moreover, this method has not been validated in pediatric populations.

The electrical activity of the diaphragm (Edi) is the myogenic potential of the diaphragmatic crura obtained by positioning a catheter in the esophagus [4]. The intensity of the Edi correlates with inspiratory effort [5]. Along with the Edi, the ratio of tidal volume to the Edi known as neuroventilatory efficiency (NVE) has been reported as an index for ventilation efficiency [6].

We report a case where the Edi and NVE measurements during spontaneous breathing trials (SBTs) were useful for determining the extubation readiness for an infant who failed primary extubation despite improvement in respiratory mechanics.

## Case presentation

A 6-month-old female (body height 65 cm, body weight 6.6 kg) with tetralogy of Fallot, pulmonary artery atresia, and major aortopulmonary collateral artery (MAPCA) underwent palliative reconstruction of the right ventricular outflow tract and unifocalization of the MAPCA. Anesthesia was induced with sevoflurane, and the trachea was intubated with an uncuffed 3.0-mm tube. After induction, anesthesia was maintained with fentanyl, midazolam, and rocuronium. A total dose of 100 mcg/kg fentanyl and 1.5 mg/kg midazolam was administered during the operation. The operation was uneventful until separation from cardiopulmonary bypass, when her oxygen saturation could not be maintained due to the presumably high resistance of the pulmonary artery. Thus, she was transferred to the ICU with the sternum open, supported by venoarterial extracorporeal membrane oxygenation (ECMO). She was weaned off ECMO on the third day of admission (day 3) to the ICU. However, she required prolonged mechanical ventilation for the following reasons. First, venoarterial ECMO support was reinitiated on day 6 because an attempt of delayed sternal closure led to severe desaturation due to pulmonary hypertensive crisis. Second, percutaneous balloon dilatation of the left pulmonary artery stenosis performed on day 13 was complicated by severe bleeding from the trachea. Furthermore, on day 28, she experienced non-obstructive mesenteric ischemia and underwent exploratory laparotomy, resulting in marked abdominal distention and generalized edema.

She was finally weaned off ECMO support on day 36. The patient was sedated with dexmedetomidine, midazolam, fentanyl, and chlorpromazine hydrochloride during treatment in the ICU, but the drug doses were gradually decreased, resulting in minimum use of sedatives on day 52 (fentanyl 0.2 mcg/kg/h, midazolam 0.03 mg/kg/h, and dexmedetomidine 0.2 mcg/kg/h). On day 52, she met the extubation criteria with a rapid shallow breathing index of 6.5 and improved dynamic Crs at 0.65 mL/cmH_2_O/kg with no clinical signs of high airway resistance. Thus, her trachea was extubated on day 52. After extubation, ventilation and oxygenation were not problematic with high flow nasal cannula support for several hours with no clinical signs of upper airway stenosis. However, she became restless with sternal retraction several hours after extubation, suggesting increased respiratory effort. The arterial blood gas analysis showed elevated pCO_2_ (53 mmHg), and she was reintubated 20 h after extubation.

### Progress from reintubation to final extubation

After reintubation, Edi monitoring was initiated to assess diaphragm function. Measurements were carried out using a 12 Fr Neurally Adjusted Ventilatory Assist (NAVA) catheter attached to a SERVO-i ventilator (Maquet Critical Care, Solna, Sweden). The catheter position was adjusted with the Edi catheter position tool equipped in the NAVA software and confirmed by daily chest X-rays. Mechanical ventilation was managed by the Synchronized Intermittent Mandatory Ventilation (SIMV) mode with pressure support (PS) and positive end-expiratory pressure (PEEP) properly applied to ensure respiratory muscle unloading. Her typical Edi values during SIMV were in the range of 10–20 mcV. Daily SBTs from 15 min to 30 min (PS 5 cmH_2_O, PEEP 5 cmH_2_O) were performed from day 53. The maximal Edi and NVE values were recorded during the SBTs (Table 1). In summary, the Edi during the SBTs was higher than 70 mcV just after the reintubation, indicating a strong inspiratory effort to deliver normal tidal volume; the Edi decreased gradually day by day while the tidal volume showed nearly constant values, leading to improvement of NVE. On day 58, her Edi values during the SBTs were 10–20 mcV, comparable to those during SIMV, and her trachea was extubated. After extubation, the patient received high-flow nasal cannula support and did not experience reintubation owing to respiratory collapse. She was discharged from the hospital without oxygen support on day 158.

**Table 1:** Measurement of Edi under SBT

Day	Edi (mcV)	TV (mL)	RR (breaths/min)	RSBI (breaths/min/mL/Kg)	NVE mL/mcV
53	70				
55	40	40	35	5.6	1.0
57	25	40	40	6.4	1.6
58	22	35	42	7.7	1.6
59	13	33	42	8.1	2.5

After reintubation, Edi and NVE were recorded during daily trials of SBT. Immediately after reintubation, Edi was higher than 70 mcV but gradually decreased to 13 mcV on day 59

*Edi* electrical activity of the diaphragm (mcV), *TV* tidal volume (mL), *RR* respiratory rate (/min), *RSBI* rapid shallow breathing index (RR/{TV/body weight}), *NVE* neural ventilatory efficiency (mL/mcV)

## Discussion

Diaphragmatic atrophy has been observed as early as 18 h after initiation of mechanical ventilation [7]. Another study showed that the pressure-generating ability of the diaphragm decreased by 30% after 5–6 days of mechanical ventilation [8]. Therefore, extreme caution should be taken for diaphragm dysfunction when extubating long-term intubated patients [9]. SBTs are commonly carried out to evaluate the readiness of extubation [10] Although the findings of numerous trials support the usefulness of SBTs for predicting extubation readiness in pediatric patients, it is nonetheless not 100% sensitive.

The Edi reflects the respiratory drive and its response to the diaphragm, suggesting its utility as an objective monitoring tool of diaphragm function [11]. Especially, NVE, the value calculated as tidal volume divided by the Edi, has been reported as an index of ventilation efficiency [12]. Again, even if tidal volume is normal, there is a possibility of extubation failure in a situation where the Edi is high and NVE is low. Therefore, in long-term intubated patients, evaluation of the Edi and NVE over time may be helpful in determining extubation readiness.

In our case, the patient required mechanical ventilation for 50 days and the presence of severe diaphragm dysfunction was expected. The first attempt of extubation was performed with successful SBTs and improvement in Crs. In addition to this, the maximal inspiratory negative pressure was − 77 cm H_2_O, which was indicative of the ability to generate sufficient pressure [13]. Indeed, immediately after extubation, the respiratory pattern of the patient was normal with acceptable arterial blood gas analysis, suggesting that the patient could be weaned from mechanical ventilation for a short time. However, these facts did not predict the ability to endure high workloads for a longer period. In this regard, Edi monitoring to assess NVE was useful to predict the fatigability of the diaphragm in this patient. Immediately after reintubation, the Edi value was higher than 70 mcV. Although the maximal Edi value varies among individuals, the Edi value higher than 70 mcV observed after reintubation in this case was indisputably high [4]. After several days of supported mechanical ventilation, the Edi values decreased while tidal volume remained constant (6–7 mL/kg), suggesting improvement of ventilatory efficiency. We finally decided to extubate the trachea of the patient on the day when the Edi during the SBTs became comparable to that during pressure support ventilation.

Moreover, the Edi refers to the myogenic potential of the diaphragmatic crura. Therefore, the Edi is not observed in fully mechanically ventilated patients. In situations when a patient does not have diaphragmatic dysfunction, weaning off from mechanical ventilation will increase tidal volume and the Edi in parallel. The optimal timing to extubate the trachea is when the tidal volume reaches acceptable values. One previous report showed that the increase in the Edi was well correlated with extubation readiness. In contrast, when patients have diaphragmatic dysfunction, the Edi and tidal volume increase with weaning from mechanical ventilation in the same manner. However, the observed Edi would reflect high effort to achieve tidal volume. If there is only information on tidal volume, clinicians might consider extubation when the tidal volume reaches an acceptable value, but this might result in extubation failure. In such cases, the Edi might be useful to assess diaphragmatic dysfunction since the Edi is expected to decrease when diaphragmatic function recovers.

Ultrasound sonography has been recently proposed for the evaluation of the structure and function of the diaphragm [14]. It is now rapidly gaining popularity with advances in image resolution. Ultrasonography is non-invasive and carries an advantage over other methods such as chest radiography or fluoroscopy. However, the method is prone to intra- and interobserver differences [15]. On the contrary, Edi and NVE values are easy to obtain with no interobserver differences. The technique also allows for real-time monitoring of breathing in clinical settings. Although each technique has its own strengths and weaknesses, the relationship between the methods should be assessed in the future study.

Several limitations should be acknowledged when interpreting this case study. First, the Edi value is affected by the catheter position and impedance of the esophagus and the thoracic cavity. Thus, normal values differ among patients [16]. Second, as mentioned previously in the “Discussion” section, it is necessary to note that the Edi may increase and extubation may be possible and vice versa. Thus, it should be emphasized that these values should be used in conjunction with the clinical findings.

In conclusion, we have experienced a case in which the Edi and NVE were valuable for deciding the extubation readiness in a long-term mechanically ventilated patient.

## Abbreviations

Crs: Compliance of the respiratory system
ECMO: Extracorporeal membrane oxygenation
Edi: Electrical activity of the diaphragm
ICU: Intensive care unit
MAPCA: Major aortopulmonary collateral artery
NAVA: Neurally Adjusted Ventilatory Assist
NVE: Neuroventilatory efficiency
PEEP: Positive end-expiratory pressure
PS: Pressure support
RR: Respiratory rate
SBT: Spontaneous breathing trial
SIMV: Synchronized Intermittent Mandatory Ventilation
TV: Tidal volume
